# Effects of adding cocoa fermentation medium on cigar leaves in agricultural fermentation stage

**DOI:** 10.3389/fbioe.2023.1251413

**Published:** 2023-08-17

**Authors:** Pengfei Zong, Wanrong Hu, Yang Huang, Hongyue An, Qianying Zhang, Zhishun Chai, Yun Kang Lei, Jun Wang, Dongliang Li, Wen Cai

**Affiliations:** ^1^ School of Materials Science and Technology, China University of Geosciences, Beijing, China; ^2^ China Tobacco Sichuan Tobacco Industry Co., Ltd., China Tobacco Technology Innovation Center for Cigar, Chengdu, China; ^3^ Sichuan Tobacco Corporation Deyang Branch, Chngdu, China

**Keywords:** cigar, agricultural fermentation, fermentation medium, chemical constituents, microbial diversity

## Abstract

**Background and Objective:** With the development of the world economy and the integration of cultures, the Chinese cigar market has shown a significant upward trend. However, high-quality cigar leaves are mostly produced in Dominica, Cuba, Nicaragua and other places. In contrast, Chinese cigar leaves have problems such as insufficient aroma, which has become one of the main factors restricting the development of Chinese cigars. Adding medium to ferment is a traditional method in the cigar industry. At present, it mostly relies on manual experience, and lacks systematic and scientific research. At the same time, the addition of medium fermentation is mainly concentrated in the industrial fermentation process, and has not yet begun to be applied in the agricultural fermentation process. In this study, the medium was added to the agricultural fermentation process for the first time to explore the possibility of the application. The effects of adding cocoa medium to ferment on the chemical composition, sensory quality and surface microbial diversity of eggplant core cigar leaves were investigated.wrapper.

**Method:** With Dexue 7′ as the experimental material, the changes of main chemical components of wrapper fermented with water and cocoa medium were determined, and microbial community structure on the surface and relative abundance of cigar leaves at different turning periods were analyzed, and the functional genes were predicted. The results of the study were as follows: 1) The results of sensory evaluation showed that the addition of cocoa medium could highlight the aroma of bean, cocoa and coffee, improve the sweetness and fluency and the combustibility of cigar leaves. 2) The addition of cocoa medium increased the contents of proline and malic acid which were positively correlated with sensory quality, and decreased the contents of citric acid, linoleic acid, basic amino acids and aromatic amino acids which were negatively correlated with sensory quality. 3) The addition of cocoa medium increased the total amount of aroma components in cigar leaves, especially carotenoid degradation products, and changed the structural composition of some aroma substances in wrappercigar leaves. 4) The similarity of species composition between the water-added group and the cocoa-added group was higher, but the dominant microorganisms were more concentrated. *Staphylococcus* and *Arthrobacter* maintained a high relative abundance throughout the fermentation process, which may be the key microorganisms in the agricultural fermentation stage. 5) The addition of cocoa medium increased the expression abundance of related functional genes in cigar leaves, accelerated the fermentation process of cigar leaves, and bacteria played a major role in the fermentation process.

**Conclusion:** Adding cocoa medium in the agricultural fermentation stage, the changes of bacterial community and dominant flora on the surface of cigar leaves are the main factors affecting their internal chemical components, and the addition of media has a positive effect on tobacco fermentation.

## 1 Introduction

Cigar is a kind of handmade products composed of cigar leaves include wrapper, binder and wrapper cigar leaves (Li et al., 2012). After the cigar leaves are harvested from the field, they need to go through the drying stage first, and agricultural fermentation is carried out after the drying. During the fermentation process, the cigar cigar leaves undergo a variety of ways such as enzyme action, chemical action and microbial action, and the internal substances are transformed. After fermentation, the color of cigar leaves becomes darker, the aroma begins to appear, the bitter taste and mixed gas will be reduced, and the color of ash begins to improve, the flammability is improved, the elasticity of cigar leaves is enhanced, and the overall quality is improved (Jin, 1998; Zhang et al., 2020). Therefore, agricultural fermentation is one of the important processes of cigar leaf production. The quality of cigar leaves in different parts of different varieties is usually different. Some cigar leaves still have some problems after agricultural fermentation. Therefore, studying the fermentation process is helpful to improve the fermentation efficiency.

Fermentation of cigar cigar leaves is usually carried out by box or stacking, and medium can be added in fermentation. In Shifang, Sichuan, China, the traditional process is to stir-fry red rice and then add water to obtain paste rice water, which is added to cigar leaves for fermentation. Studies have shown that paste rice juice has a very significant inhibitory effect on aerobic bacteria and anaerobic bacteria in cigar leaves. The more the amount of paste rice juice, the more obvious the inhibitory effect. Rice paste itself has no obvious inhibitory effect on mold and yeast, but it shows the effect of inhibiting mold and yeast in the process of stacking solid-state fermentation (Li et al., 2016). Li Ling found that rice wine treatment could increase the content of solanone and browning products in cigar leaves. Compared with the control group, the quality of cigar leaves was improved significantly, and different excipients were added during fermentation, such as extract of green tea, diluted rice wine and diluted pure milk, which could improve the quality of cigar leaves from different aspects (Li et al., 2016; Ling and Zhang, 2016). Li Linlin et al. used the enzymatic hydrolysate of cigar powder as the reaction base liquid, and added different amino acids and carbohydrate combinations to carry out the Maillard reaction. Adding the medium to cigar cigar leaves can promote the formation of aroma substances, and most of the flavor substances that contribute to the flavor characteristics of cigar cigar leaves after different treatments have increased to varying degrees, which can better promote the manifestation of cigar style and increase the aroma richness of cigar (Li et al., 2019). Citric acid, white sugar, cocoa powder, jujube juice, wine juice, holly glue and so on are some common used fermentation media. Previous studies have shown that adding medium to participate in fermentation can significantly improve the quality of cigar leaves. The nicotine content, bitter taste and irritation in cigar leaves are significantly reduced, which can improve the maturity and proficiency of cigarcigar leaves to a certain extent (Hu et al., 2023; Yin et al., 2023). At present, in the cigar industry, adding medium for fermentation is a hot spot in the industry. Cigars are handmade products with low quality stability. The quality of cigar is mainly related to tobacco leaf. The quality of cigar leaves is related to cultivation and fermentation measures. Adding medium for fermentation can improve the quality of tobacco leaf. At present, in industrial production, China Tobacco Sichuan Industrial Co., Ltd. has begun to add medium for fermentation in the industrial fermentation stage, and the research on adding medium in the agricultural fermentation stage is still in its infancy. The reason why cigar production needs industrial fermentation is that agricultural fermentation is not sufficient, which can not solve the problems of poor quality of cigar leaves and heavy miscellaneous gas. If these problems can be solved in the agricultural fermentation process by adding medium for fermentation, the production cost of the enterprise will be greatly saved.In this study, the cocoa medium was taken as an example to study the effect of adding medium on the quality of cigar cigar leaves in the agricultural fermentation stage, so as to reveal the mechanism of cocoa medium in the agricultural fermentation process.

## 2 Materials and methods

### 2.1 Materials

Cigar leaves: the middle second grade of Dexue 7 wrapperwrapper cigar leaves produced in Shifang, Sichuan harvesting in 2019.

DNeasy PowerSoil Kit, QIAamp 96 PowerFecal QI-Acube HT Kit: QIAGEN, United States; qubitds deoxyribonucleic acid (DNA) Assay Kit: Life Technologies Inc., United States; tks Gflex DNA polymerase (1.25 U/μL): Dalian Takara Company. Acetonitrile (chromatographically pure, J.T. Baker, United States).

Cocoa medium solution: 20 g cocoa powder was dissolved in 150 mL water, centrifuged at 500 rpm/min, and the supernatant was taken for standby. When added to the cigar leaves, the cocoa medium is mixed well to avoid the material precipitation at the bottom.

EL204 Electronic Balance, METTLER TOLEDO Instruments (Shanghai) Co., Ltd.; agilent High Performance Liquid Chromatograph, Agilent, United States; TND03-H Mixing type dry thermostat, Shenzhen Tuonengda Technology Co., Ltd.; Automatic fat analyzer, Haineng Instrument Co., Ltd.; SH420F graphite digestion instrument, Haineng Scientific Instrument Co., Ltd.; ETC., 811, PCR amplifier, Beijing Dongsheng Innovation Biotechnology Co., Ltd.; Haineng K9860 automatic Kjeldahl nitrogen analyzer, Haineng Scientific Instrument Co., Ltd. ; Nexis GC-2030 gas chromatograph, Shimadzu, Japan. (Wen et al., 2022).

### 2.2 Methods

#### 2.2.1 Fermentation of wrapper

The moisture content of cigar leaves before agricultural fermentation is generally about 20%, and it is necessary to add water to resurge the cigar leaves. The moisture content of cigar leaves after resurgence is about 26%. The specific operation steps are as follows: untie a handful of cigar leaves and spread them on the table, use an electronic sprayer to evenly spray the medium solution on the surface of the cigar leaves. After half of the spraying, turn the cigar leaves over and continue to spray the other side to ensure uniform spraying. After the spraying is completed, the cigar leaves are suspended, and the water is naturally absorbed and tied. Pile the tied cigar leaves to the tray, and pile the tray into a large stack. Tray specifications, 1 m long, 1.2 m wide. In general, a pile of fermented wrapper cigar leaves is about 2000 kg, 1.5–1.8 m high, 2–2.4 m wide, and the length increases from 3.6 m. The temperature in the pile of cigar leaves was controlled between 32°C and 40°C.During the fermentation, the temperature in the pile was observed in time. When the core temperature of stack reached the preset maximum temperature about 45°C, the pile was turned in time. Turn the reactor in time when the core temperature does not rise to the preset maximum and does not rise for 2–3 days or when the core temperature drops slightly. 3 handfuls of cigar leaves were extracted at each turning point (upper, middle and lower), and 100 g of cigar leaves were sampled for each handfuls. In this experiment, the pile was turned twice, and the cigar leaves were broken and mixed after each sample for detection.

#### 2.2.2 Sensory quality evaluation

Cigar leaves with a diameter of 38 rings and a length of 110 mm were rolled into cigarettes, which were placed in a constant temperature and humidity box at 20°C and 65% relative humidity to balance water for 30 days. Seven people with sensory evaluation qualifications were organized to conduct evaluation according to YC/T415-2011 (Shen et al., 2011).

#### 2.2.3 Detection of conventional chemical components of tobacco

In this experiment, continuous flow analyzer was used to determine the content of conventional chemical components of tobacco, including total sugar, reducing sugar, alkaloid, total nitrogen, potassium, chlorine and other indicators. Refer to YC/T 159-2002, YC/T 160-2002, YC/T 161-2002, YC/T 162-2002, YC/T 217-2007 and YC/T 216-2007 for details (Yan et al., 2015).

#### 2.2.4 Determination of non-volatile organic acid content

The content of non-volatile organic acids was determined by gas chromatography according to the method in reference (Liu et al., 2014).

#### 2.2.5 Determination of amino

Amino acid content was determined by YCT 448–2012 determination of free amino acids in tobacco and tobacco products-ion chromatography-integrated pulse amperometric method (Wang et al., 2009; Yu et al., 2022).

#### 2.2.6 Determination of flavor substances

Sample pretreatment: About 1 g of crushed tobacco samples were weighed and placed in a 22 mL headspace bottle, which was sealed by a headspace bottle cap, and the samples were extracted by a manual solid-phase microextraction device. An automated headspace solid-phase microextraction (HS-SPME) device equipped with 50/30 μm DVB/CAR/PDMS fibers was exposed to the top space of the sample bottle and extracted for 30 min at a speed of 20 mm/s at 60°C. After extraction, the GC analyzer was desorbed in a splitless mode at 250°C for 1 min, and the volatile substances were determined by GC-MS (Wu, 2012).

GC conditions: RTX-waxMS chromatographic column, flow rate 1.0 mL/min, helium as carrier gas, inlet temperature 250. The temperature programming conditions were as follows: 60°C for 2 min, 10°C/min to 110°C, 3°C/min to 150°C, 15°C/min to 230°C, 20 min.

MS conditions: electron impact ion source (EI), ion source temperature 230°C, quadrupole temperature 150°C, transmission line temperature 230°C; electron energy:70 eV, electron multiplier voltage:1500 V; the scanning quality range was 45–350 m/z.

The identification of volatile compounds was based on the comparative analysis of computer spectral library WILEY 8.0 and NIST 14. Only the identification results with positive and negative similarities greater than 800 were reported, and the relative content was calculated by area normalization method.

#### 2.2.7 Determination of microbial diversity

Total bacterial DNA was extracted according to the instructions of E.Z.N.A. ^®^ soil DNA kit (Omega Bio-tek, Norcross, GA, United States). The quality of DNA extraction was detected by 1% agarose gel electrophoresis, and the concentration and purity of DNA were determined by NanoDrop 2000. The V3-V4 region of 16S rRNA gene was amplified by PCR using 338F (5' -ACT​CCT​ACG​GGA​GGC​AGC​AG-3 ′) and 806R (5' -GACTACHVGGGTWTCTAAT-3 ′). The ITS11-1F region of FunglITS gene was amplified by PCR using ITS1-1F-F (CTT​GGT​CAT​TTA​GAG​GAA​GTA​A) and ITS1-1F-R (GCT​GCG​TTC​TTC​ATC​GAT​GC). PCR products were recovered by 2% agarose gel, purified by AxyPrep DNA Gel Extraction Kit (Axygen Biosciences, Union City, CA, United States), and quantified by Quantus TM Fluorometer. The amplified sequence library was established using NEXTFLEX Rapid DNA-Seq Kit (New England Biolabs Inc., Ipswich, 127 MA, United States), and then high-throughput sequencing was performed using the Illumina Miseq PE300 sequencing platform (Illumina Corporation, San Diego, United States). FLASH software was used to splice the original sequence, and USEARCH software (version 7.0) was used to cluster the sequence and eliminate the chimeric sequence. The dilution curve, species composition and abundance distribution table were calculated, and the functional genes of the sample colonies were predicted by PICRUSt2 and FUNGuild methods.

#### 2.2.8 Data analysis

SPSS statistical software was used for data analysis, and data mapping was performed by Origin software. The grouping of samples is shown in Table 1.

**TABLE 1:** Sample grouping table.

Sample	Group	Notes
KKNYF_01	A	Control/unfermented sample/parallel 1
KKNYF_02	A	Control/unfermented sample/parallel 2
KKNYF_03	A	Control/unfermented sample/parallel 3
KKNYF_11	B	Fermentation with water/turned over the heap for the first time/parallel 1
KKNYF_12	B	Fermentation with water/turned over the heap for the first time/parallel 2
KKNYF_13	B	Fermentation with water/turned over the heap for the first time/parallel 3
KKNYF_21	C	Fermentation with cocoa/turned over the heap for the first time/parallel 1
KKNYF_22	C	Fermentation with cocoa/turned over the heap for the first time/parallel 2
KKNYF_23	C	Fermentation with cocoa/turned over the heap for the first time/parallel 3
KKNYF_31	D	Fermentation with water/turned over the heap for the second time/parallel 1
KKNYF_32	D	Fermentation with water/turned over the heap for the second time/parallel 2
KKNYF_33	D	Fermentation with water/turned over the heap for the second time/parallel 3
KKNYF_41	E	Fermentation with cocoa/turned over the heap for the second time/parallel 1
KKNYF_42	E	Fermentation with cocoa/turned over the heap for the second time/parallel 2
KKNYF_43	E	Fermentation with cocoa/turned over the heap for the second time/parallel 3
KKNYF_51	F	Fermentation with water/end of fermentation/parallel 1
KKNYF_52	F	Fermentation with water/end of fermentation/parallel 2
KKNYF_53	F	Fermentation with water/end of fermentation/parallel 3
KKNYF_61	G	Fermentation with cocoa/end of fermentation/parallel 1
KKNYF_62	G	Fermentation with cocoa/end of fermentation/parallel 2
KKNYF_63	G	Fermentation with cocoa/end of fermentation/parallel 3

## 3 Results

### 3.1 Sensory evaluation

From the results of sensory evaluation, the original sample had a heavy dry, green and protein flavor, poor smoke fullness, fluency and fineness, and general flammability. After the fermentation, the mixed gas in the water-added group was lower than that in the blank group, and the aroma richness and aroma volume were improved. The smoke was soft and delicate, the sweetness was better, the fluency was increased, the maturity was enhanced, and the flammability was better. In the cocoa medium fermentation group, the aroma of beans, coffee and cocoa was prominent, the mixed gas was significantly reduced, the aroma was rich, the smoke was soft and delicate, the sweetness was good, the fluency was increased, the maturity was enhanced, the combustion was good, and the quality characteristics were significantly improved. The sensory evaluation results of each group of samples are shown in Table 2.

**TABLE 2:** Results of sensory evaluation.

Sample	Style	Concentration	Flavor note	Miscellaneous gas	Quality characteristic
A	More significant	Above medium	Bean, nuts, coffee, burnt sweet flavor, slightly with baking and hay flavor	With a scorched, green and protein smell	The aroma is sufficient, rich, irritating, residual, flue gas fluency and fineness is poor, the combustion is general
B	More significant	Above medium	Bean, nuts, coffee, burnt sweet flavor, slightly with baking and hay flavor	The aroma of green miscellaneous, dry coke and protein decreased slightly	Aroma is sufficient, rich, general combustion
C	More significant	Above medium	Bean, nuts, coffee, burnt sweet aroma, cocoa aroma highlights	The coke is slightly heavier and the protein smell is obvious	Aroma is sufficient, rich, general combustion
D	More significant	Above medium	Bean, nuts, coffee, burnt sweet aroma, burnt sweet and bean aroma increased	The coke gas is slightly heavy, the protein breath is obvious, and the green gas is slightly reduced	The aroma is sufficient, rich, and the flammability is slightly improved
E	More significant	Above medium	Bean, nuts, coffee, burnt sweet aroma, cocoa and bean aroma increased	The coke gas and green gas decreased	The softness and fluency of smoke increased, the irritation decreased, and the combustibility improved slightly
F	More significant	Above medium	Bean, nuts, coffee, cocoa, burnt sweet aroma, supplemented by baking and hay	Slightly burnt and protein	The aroma is rich, the smoke is soft and delicate, the sweetness is good, the maturity aftertaste is increased, and the flammability is good
G	More significant	Above medium	Bean, nuts, coffee, cocoa, burnt sweet flavor, beans, coffee, cocoa flavor prominent	Miscellaneous gas significantly reduced, slightly protein breath	The aroma is rich, the smoke is soft and delicate, the sweetness is good, the fluency is increased, the maturity aftertaste is enhanced, and the flammability is good

### 3.2 Conventional chemical composition analysis

**FIGURE 1:**
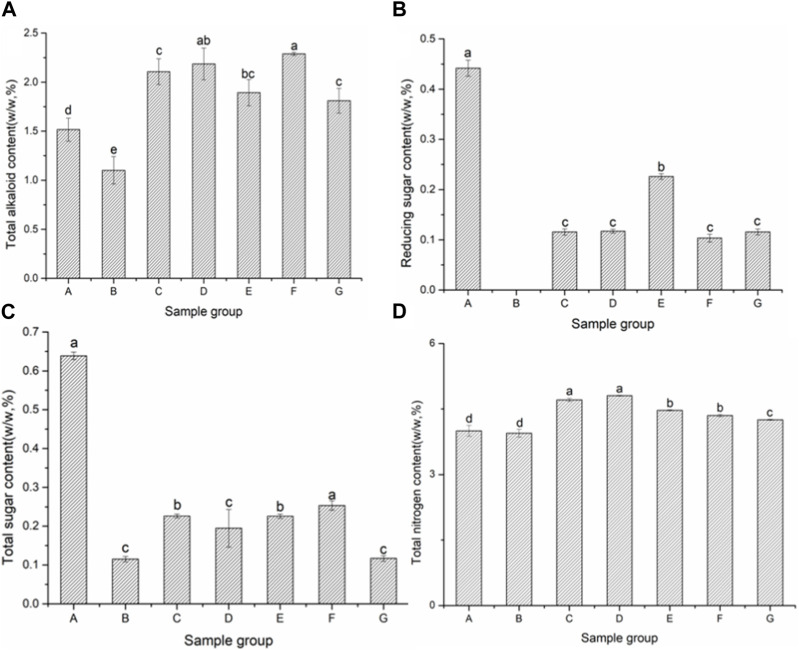
Conventional chemical constituents of cigar cigar leaves. Note: Different lowercase letters indicate that the difference of samples in control-group at different fermentation stages is statistically significant at *p* < 0.05 level. Different capital letters indicate that the difference of samples in control-group at different fermentation stages is statistically significant at *p* < 0.05 level, **(A)** is the total alkaloid content, **(B)** is the reducing sugar content, **(C)** is the total sugar content, **(D)** is the total nitrogen content.

It can be seen from Figure 1A that the content of total alkaloids in the cocoa medium fermentation group was significantly higher than that in the blank control group but slightly lower than that in the water fermentation group, which may be due to the slightly higher microbial activity in the medium fermentation group, prompting more alkaloids to degrade under the action of enzymes, microorganisms and oxygen. The main degradation products are nicotinic acid, 3-pyridylacetone, etc., which contribute to the aroma of cigar leaves (Zhao et al., 2012; Rong et al., 2021). Figure 1B shows the changes of reducing sugar in cigar leaves, and there was no significant difference in the changes of samples in each group (*p* > 0.05). Figure 1C shows the changes of total sugar content in cigar leaves, and there was significant difference between each group (*p* < 0.05). At the end of fermentation, the total sugar content in the medium fermentation group was significantly lower than that in the water fermentation group, and it was also possible that the higher microbial activity in the medium fermentation group affected the total sugar consumption.

Studies have shown that the flammability of cigar leaves is related to potassium, chlorine content and potassium-chlorine ratio, and potassium content and potassium-chlorine ratio are positively correlated with the flammability of cigar leaves (Hu et al., 2021). As shown in Table 3, at the end of fermentation, compared with the control group, the decrease of potassium content and potassium chloride in the water-added group was more obvious than that in the cocoa-added group, which may be due to the dilution of potassium and chlorine content in cigar leaves after adding water. At the end of fermentation, the potassium chloride ratio in the cocoa-added group was 13.33% higher than that in the water-added group, and the potassium content was 18.73% higher than that in the water-added group, which was consistent with the evaluation of combustibility in the sensory evaluation results, indicating that the addition of cocoa medium fermentation can improve the combustibility of cigar leaves to a certain extent.

**TABLE 3:** Sample potassium chloride ion content and potassium chloride ratio.

样本分组	K (w/w, %)	Cl (%, w/w)	K/Cl (w/w)
A	5.928 ± 0.054a	0.225 ± 0.013d	26.322 ± 1.77a
B	5.236 ± 0.118c	0.326 ± 0.017c	16.067 ± 0.485b
C	4.592 ± 0.0169d	0.476 ± 0.013a	9.646 ± 0.314e
D	5.445 ± 0.120bc	0.463 ± 0.017a	11.749 ± 0.187d
E	5.475 ± 0.0926d	0.371 ± 0.008b	14.730 ± 0.103b
F	4.485 ± 0.134d	0.351 ± 0.014bc	12.760 ± 0.154cd
G	5.325 ± 0.049b	0.368 ± 0.005b	14.462 ± 0.342bc

Note: Different English letters indicated that the difference of samples in each group was statistically significant at *p* < 0.05 level.

### 3.3 Effect of medium fermentation on non-volatile organic acid content

The organic acids in cigar leaves can be divided into volatile and non-volatile. The relative content of dibasic acid and tribasic acid in non-volatile organic acids is relatively high, and the content of malic acid, citric acid and oxalic acid is relatively high (Wang et al., 2009). Non-volatile organic acids have no significant direct effect on the aroma of cigarette smoke, but they can synthesize salts with alkaloids, adjust the ratio of protonation and free nicotine, adjust the pH of cigarettes, increase the concentration of smoke, improve the taste, and make the smoke become mellow, indirectly affect the smoke aroma of cigar leaves, and play a balancing role in smoke. Malic acid can increase the acidity of flue gas, improve the characteristics of flue gas, especially for tobacco with high alkaloid content, and make the flue gas calm. Citric acid content was negatively correlated with tobacco quality, resulting in poor taste. The content of unsaturated fatty acids such as linoleic acid and linolenic acid is negatively correlated with the aroma and taste of cigar leaves, because they can increase the roughness, irritation and green odor of flue gas, and produce astringency (Zhang et al., 2012). It can be seen from Table 4 that after the fermentation, the malic acid content in the cigar leaves fermented with cocoa increased by 9.69%, the citric acid content decreased by 3.18%, and the linoleic acid content decreased by 5.77%. Studies have shown that the addition of cocoa medium can increase the content of cigar malic acid to a certain extent, reduce the content of non-volatile organic acids that are negatively correlated with the quality of cigar leaves, and improve the quality of cigar leaves.

**TABLE 4:** Content of non-volatile organic acids in petroleum ether extract of cigar leaves.

Nonvolatile organic acids(ug/g)/Sample	A	B	C	D	E	F	G
Adipic acid	1887.73 ± 4.92a	1840.24 ± 10.62a	1901.83 ± 1.21b	1746.43 ± 43.21d	1492.03 ± 3.59c	1757.28 ± 4.50c	1713.30 ± 19.49ac
Propionic acid	228.56 ± 2.60b	218.96 ± 0.92c	248.44 ± 0.00a	166.26 ± 2.94d	195.51 ± 0.26d	220.77 ± 0.02a	211.93 ± 0.36a
Levulinic acid	123.27 ± 1.04bc	14.98 ± 0.12c	45.68 ± 1.86a	27.01 ± 1.58b	55.92 ± 1.59b	21.26 ± 0.12c	25.83 ± 0.30bc
Succinic acid	38.97 ± 0.04c	38.55 ± 0.65d	42.60 ± 0.54a	49.52 ± 1.29c	35.72 ± 0.24b	41.65 ± 0.17a	41.50 ± 2.54 ab
Malic acid	3514.72 ± 38.96a	2602.35 ± 374.40c	2970.67 ± 42.64d	2049.42 ± 800.71a	2293.35 ± 113.49a	2090.33 ± 58.13bc	2292.89 ± 35.60ac
Citric acid	4706.78 ± 76.35b	5657.78 ± 196.53a	4051.21 ± 1.31c	5630.96 ± 31.70a	3211.19 ± 10.05c	4544.12 ± 80.15cd	4399.40 ± 61.99ae
Vanillic acid	7.79 ± 0.29c	7.22 ± 1.81ac	12.28 ± 1.81a	7.90 ± 0.11ac	7.90 ± 0.97e	8.55 ± 0.28e	8.74 ± 0.25bc
Tetradecanoic acid	10.82 ± 0.77c	8.81 ± 2.43c	12.49 ± 0.63c	8.33 ± 0.76bc	9.31 ± 1.12e	12.50 ± 3.04bc	10.14 ± 1.43b
Hexadecanoic acid	80.50 ± 1.96a	61.40 ± 1.85bc	85.01 ± 0.06b	80.41 ± 0.88bc	77.60 ± 0.08e	64.25 ± 1.69c	76.85 ± 10.47bc
Linoleic acid	48.46 ± 1.82e	34.72 ± 0.54ac	56.34 ± 1.13c	54.70 ± 0.20a	50.26 ± 1.12e	46.41 ± 0.29d	43.73 ± 2.63b
Octadecanoic acid	25.73 ± 0.70e	22.36 ± 0.93bc	37.01 ± 0.27c	26.34 ± 3.35 ab	26.66 ± 0.63d	21.72 ± 0.22ce	24.88 ± 3.97e
Teicosanoic acid	10.45 ± 1.03a	8.73 ± 1.37a	11.61 ± 2.78c	9.92 ± 0.90b	9.21 ± 0.07c	9.42 ± 1.41d	22.51 ± 19.39b

Note: Different English letters indicated that the difference of samples in each group was statistically significant at *p* < 0.05 level.

### 3.4 Effect of medium fermentation on amino acid content

The results showed that there were some differences in the sensory quality of cigar leaves among different components of amino acids in Table 5. The content of most amino acids except proline and its proportion in the total free amino acids were negatively correlated with the aroma quality, taste comfort, color purity and oil content of cigar leaves, while the proportion of proline in the total free amino acids was positively correlated with the quality of cigar leaves (Xu et al., 2020). The basic amino acids were mainly lysine (Lys), arginine (Arg), histidine (His), and the aromatic amino acids were mainly tyrosine (Tyr), phenylalanine (Phe) and tryptophan (Try). The total amount of basic amino acids and aromatic amino acids in the water-added group was 1.840 mg/g, and the proline (Pro) content was 0.275 mg/g. The total amount of basic amino acids and aromatic amino acids in the cocoa medium fermentation group was 1.577 mg/g, and the proline content was 0.334 mg/g. The results showed that the addition of cocoa medium could reduce the basic amino acids and aromatic amino acids in cigar leaves, increase the proline content, and thus improve the sensory quality of cigar leaves.

**TABLE 5:** Amino acid composition of cigar leaves.

Amino acid content(mg/g)/Sample	A	B	C	D	E	F	G
Asp	3.199 ± 0.032a	2.672 ± 0.010b	3.173 ± 0.234b	3.312 ± 0.573c	2.522 ± 0.592e	2.821 ± 0.251d	3.137 ± 0.25ac
Glu	1.924 ± 0.213a	1.272 ± 0.225bc	2.255 ± 0.236c	2.314 ± 0.249d	2.044 ± 0.256c	1.835 ± 0.155c	1.903 ± 0.144e
Ser	0.423 ± 0.002bc	0.396 ± 0.003b	0.422 ± 0.114a	0.380 ± 0.023ac	0.446 ± 0.056c	0.415 ± 0.014c	0.403 ± 0.020e
His	0.243 ± 0.002bd	0.285 ± 0.009b	0.323 ± 0.011b	0.417 ± 0.014c	0.332 ± 0.032e	0.377 ± 0.011a	0.245 ± 0.078c
Gly	0.579 ± 0.032d	0.578 ± 0.045b	0.527 ± 0.036bc	0.516 ± 0.07e	0.617 ± 0.036c	0.543 ± 0.015d	0.549 ± 0.026d
Thr	0.411 ± 0.003e	0.420 ± 0.102de	0.406 ± 0.013ac	0.403 ± 0.023a	0.484 ± 0.056ac	0.412 ± 0.028c	0.363 ± 0.003bc
Arg	0.428 ± 0.022c	0.292 ± 0.013ce	0.363 ± 0.021ae	0.380 ± 0.022a	0.542 ± 0.069b	0.322 ± 0.047b	0.301 ± 0.002e
Ala	0.537 ± 0.001ac	0.421 ± 0.020ca	0.424 ± 0.020ce	0.427 ± 0.003 ab	0.509 ± 0.001c	0.382 ± 0.005e	0.404 ± 0.056d
Tyr	0.302 ± 0.002 ab	0.207 ± 0.011a	0.197 ± 0.003a	0.224 ± 0.026a	0.292 ± 0.036d	0.283 ± 0.021c	0.181 ± 0.004b
Cys-s	0.015 ± 0.001be	0.039 ± 0.001a	0.018 ± 0.002a	0.044 ± 0.001	0.017 ± .0004	0.024 ± 0.006	0.066 ± 0.010b
Val	0.670 ± 0.002d	0.647 ± 0.002bc	0.596 ± 0.003ac	0.616 ± 0.001c	0.713 ± 0.001b	0.576 ± 0.002e	0.595 ± 0.001c
Met	0.136 ± 0.003cd	0.156 ± 0.002b	0.174 ± 0.001ce	0.122 ± 0.001a	0.142 ± 0.002b	0.139 ± 0.002ce	0.095 ± 0.001e
Phe	0.533 ± 0.002ae	0.511 ± 0.002ac	0.542 ± 0.014c	0.610 ± 0.023ce	0.632 ± 0.032a	0.516 ± 0.048d	0.466 ± 0.001e
Ile	0.385 ± 0.001ac	0.430 ± 0.002c	0.348 ± 0.002c	0.367 ± 0.001a	0.442 ± 0.001c	0.400 ± 0.001d	0.342 ± 0.014e
Leu	0.695 ± 0.023a	0.681 ± 0.014c	0.685 ± 0.018b	0.258 ± 0.045ce	0.436 ± 0.039d	0.526 ± 0.029ce	0.586 ± 0.025a
Lys	0.427 ± 0.002bd	0.390 ± 0.003e	0.396 ± 0.005ad	0.378 ± 0.069a	0.496 ± 0.026b	0.342 ± 0.069b	0.384 ± 0.099a
Pro	0.614 ± 0.014cd	0.318 ± 0.055d	0.349 ± 0.008d	0.366 ± 0.009a	0.394 ± 0.009ac	0.275 ± 0.014ad	0.334 ± 0.056bd
Overall amount	11.521 ± 0.369ce	9.715 ± 1.001a	11.198 ± 0.036e	11.134 ± 0.014a	11.06 ± 0.068b	10.188 ± 1.002c	10.354 ± 1.003e

Note: Different English letters indicated that the difference of samples in each group was statistically significant at *p* < 0.05 level.

### 3.5 Analysis of aroma components

**FIGURE 2:**
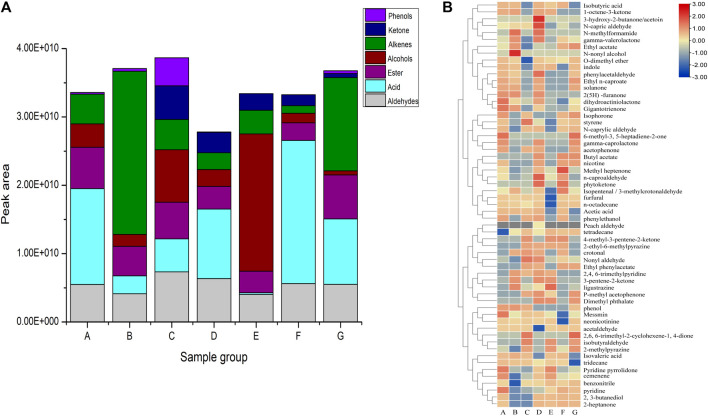
**(A)** is the Classification data of total content of aromatic ingredients, **(B)** is Heat map of some main odorant ingredients.

The aroma components in cigar leaves are directly related to the sensory quality of cigar leaves (Zhu et al., 2020; Li et al., 2012).In this study, a variety of aroma components were detected in the above cigar tobacco leaf samples. The effect of agricultural fermentation on the content of aroma components in cigar leaves is shown in Figure 2. It can be seen from Figure 2A that after the first turning, the total amount of aroma components in the water-added group was lower than that in the cocoa-added group. After the second turning, the aroma components in the two samples were mainly acids, and the total amount decreased to varying degrees. The total amount of the cocoa-added group was still higher than that of the water-added group, which may be due to the biological effects of microorganisms during this period. At the end of fermentation, the total amount of aroma components in the water-added group was significantly lower than that in the cocoa-added group. It can be seen from Figure 2B that in the cocoa-added group, the content of solanone and carotenoid degradation products megastigmatrienone, dihydroartemisinin, isophorone and other substances increased significantly, and these substances were reported to be positively correlated with tobacco aroma. This shows that the addition of cocoa medium has a significant effect on improving the content of aroma components in cigar leaves. At the same time, the main aroma components in the cocoa-added group were mainly alkenes, and the main aroma components in the water-added were mainly acids. It can be inferred that the addition of cocoa medium changed the structure of some aroma substances in cigar leaves to a certain extent.

### 3.6 Effect of cocoa medium on microbial diversity on the surface of cigar leaves

#### 3.6.1 Alpha diversity analysis

**FIGURE 3:**
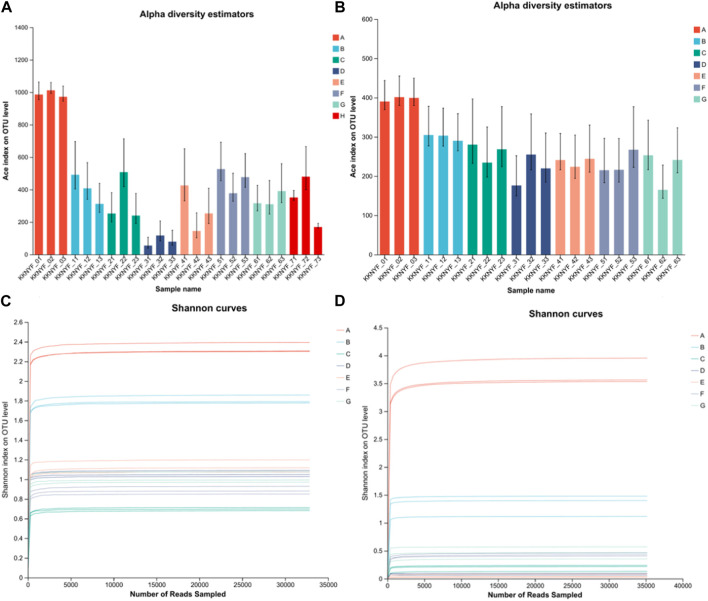
**(A)** is the bacterial diversity index map, **(B)** is the bacterial dilution curve, **(C)** is the fungal diversity index map, and **(D)** is the fungal dilution curve. Alpha diversity analysis.

The microbial diversity of cigar leaf samples was sequenced by 16S rRNA high-throughput sequencing. Through the analysis of diversity index, the abundance, coverage and diversity of species in the community can be obtained. The diversity and richness of bacteria and fungi in the samples were evaluated by Chao1, Shannon, Simpson and Coverage indexes in Alpha diversity index. The microbial coverage index of all samples in Table 6 is greater than 0.99, indicating that the sequence detection in the sample library is basically covered, and the sample sequencing can reflect the real situation of the sample. Figures 3A, B are used to illustrate the number of samples contained at the OUT level, which can characterize the diversity. From the diagram, it can be seen that the OUT diversity of unfermented tobacco samples is the highest. Dilution curve is mainly used to characterize the sequencing depth of samples, which can reflect the rationality of sequencing data. As shown in Figures 3C, D, the dilution curve based on the Shannon diversity index finally flattens out with the increase of the number of sample sequences, indicating that most of the diversity has been generated.

**TABLE 6:** Alpha diversity index statistics.

Sample	Shannon	Chao1	Coverage	Shannon	Chao1	Coverage
	(Bacteria)	(Bacteria)	(Bacteria)	(Fungus)	(Fungus)	(Fungus)
KKNYF_01	3.563	971.058	0.995	2.391	385.220	0.999
KKNYF_02	3.953	1000.148	0.996	2.316	398.439	0.999
KKNYF_03	3.535	957.322	0.995	2.315	397.000	0.999
KKNYF_11	1.117	312.813	0.998	1.799	299.083	0.999
KKNYF_12	1.401	296.214	0.998	1.791	281.065	0.999
KKNYF_13	1.477	239.313	0.998	1.872	298.000	0.999
KKNYF_21	0.131	200.214	0.998	1.031	178.368	0.999
KKNYF_22	0.237	338.000	0.998	1.096	249.231	0.999
KKNYF_23	0.218	207.500	0.998	1.050	167.273	0.999
KKNYF_31	0.065	47.000	1.000	0.849	175.000	0.999
KKNYF_32	0.093	91.500	0.999	0.927	158.625	0.999
KKNYF_33	0.094	50.143	1.000	0.889	190.833	0.999
KKNYF_41	0.081	262.100	0.998	0.677	201.059	0.999
KKNYF_42	0.031	78.429	0.999	0.724	196.200	0.999
KKNYF_43	0.038	105.000	0.999	0.710	225.214	0.999
KKNYF_51	0.460	394.667	0.997	1.196	227.406	0.999
KKNYF_52	0.428	355.727	0.997	1.121	215.385	0.999
KKNYF_53	0.405	319.319	0.997	1.072	194.435	0.999
KKNYF_61	0.354	231.903	0.998	0.975	193.955	0.999
KKNYF_62	0.466	232.091	0.998	0.995	165.882	0.999
KKNYF_63	0.570	304.462	0.998	1.082	184.966	0.999

Chao1 index and Shannon index reflected the richness and diversity of microbial community, respectively. It can be seen from Table 5 that the Chao1 index of bacteria in the control group was between 957.322-1000.148, and that of fungi was between 385.220-398.439.After the beginning of agricultural fermentation, the bacterial Chao1 index, the overall change range of the water-added group was between 47.000-394.667, and the overall change range of the cocoa-added group was between 78.429-338.000. The lowest value appeared in the water-added group, which was the lowest value at the first turning, and the fluctuation range was large. The overall fluctuation of the Chao1 index value of fungi smaller than that of bacteria.

#### 3.6.2 OTU analysis of microbial community

**FIGURE 4:**
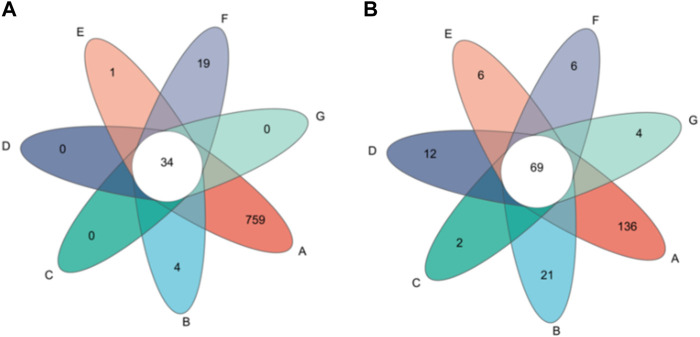
**(A)** is the bacterial OUT distribution in the sample, and **(B)** is the fungal OUT distribution in the sample. Venn diagram based on OTU of microbial community in cigar leaves.

Venn diagram was used to represent the number of common and unique OTU in the fermentation process, which could directly reflect the similarity and coincidence between samples. As shown in Figure 4, a total of 817 effective bacterial OTUs were detected in all samples. The number of unique OTUs detected respectively at the first turning time, the second turning time and the end of fermentation with water was 4,0 and 19. The number of unique OTUs detected respectively at the first turning time, the second turning time and the end of fermentation with cocoa was 0,1,0. A total of 243 effective fungal OTUs were detected in all samples. A total of 94 effective OUTs were detected in the control group. The number of unique OTUs detected at the first turning time, the second turning time and the end of fermentation with water was 17,8 and 6. The number of unique OTUs detected at the first turning, the second turning and the end of fermentation with cocoa was 1,5 and 2. The similarity of species composition between the water-added and the cocoa-added group was very high.

#### 3.6.3 Microbiological community analysis

**FIGURE 5:**
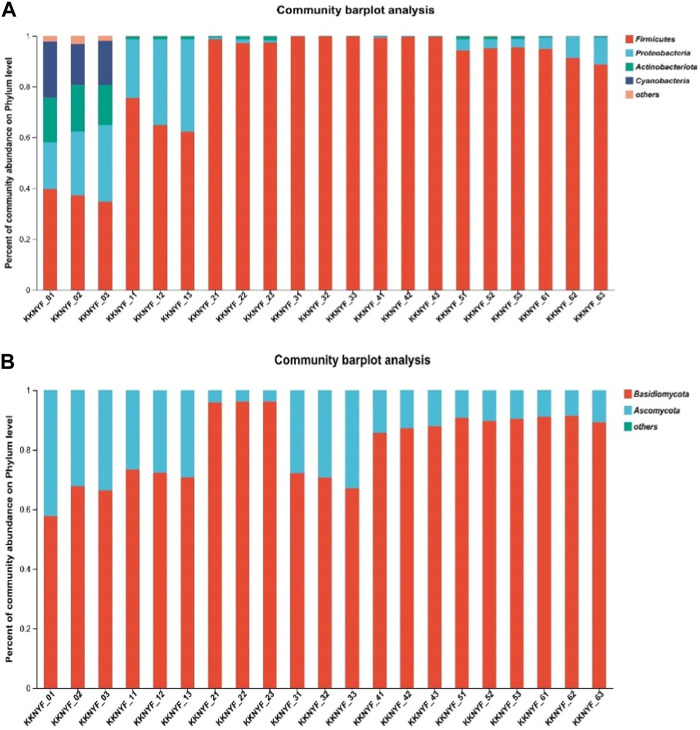
**(A)** is the relative abundance of bacteria in cigar cigar leaves, and **(B)** is the relative abundance of fungi in cigar cigar leaves. Relative abundance at phylum level.

At the phylum level, there were 4 bacterial phylums and 2 fungal phylums in the phylums with relative content greater than 1% (Figures 5A, B). Specifically, the bacterial phylum is mainly *Proteobacteria*、*Cyanobacteria*、*Firmicutes* and *Actinobacteria*, and the fungal phylum is mainly *Basidiomycota* and *Ascomycota*. The main bacterial phyla in the control group were *Firmicutes* and *Actinobacteria*, with relative abundances of 34.72% and 30.16%. In the water-added group, the dominant bacterial phylum was Firmicutes at the first turning, the second turning and the end of fermentation, and the relative abundance was 62.30%, 99.93% and 94.25%, respectively. The dominant bacterial phylum in the cocoa-added group was *Firmicutes* at the first turning time, the second turning time and the end of fermentation, and the relative abundance was 98.70%, 99.26% and 94.95% respectively.

**FIGURE 6:**
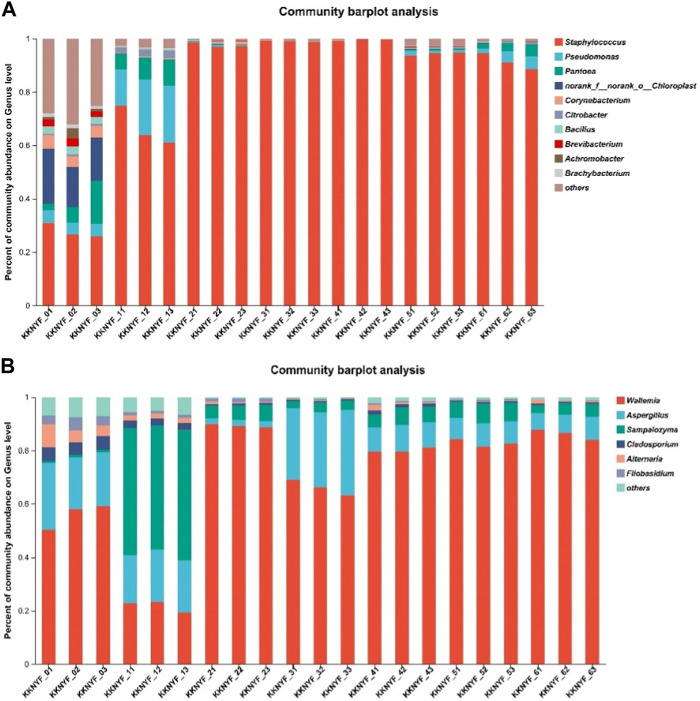
**(A)** is the relative abundance of bacteria in cigar cigar leaves, and **(B)** is the relative abundance of fungi in cigar cigar leaves. Relative abundance at genus level.

**FIGURE 7:**
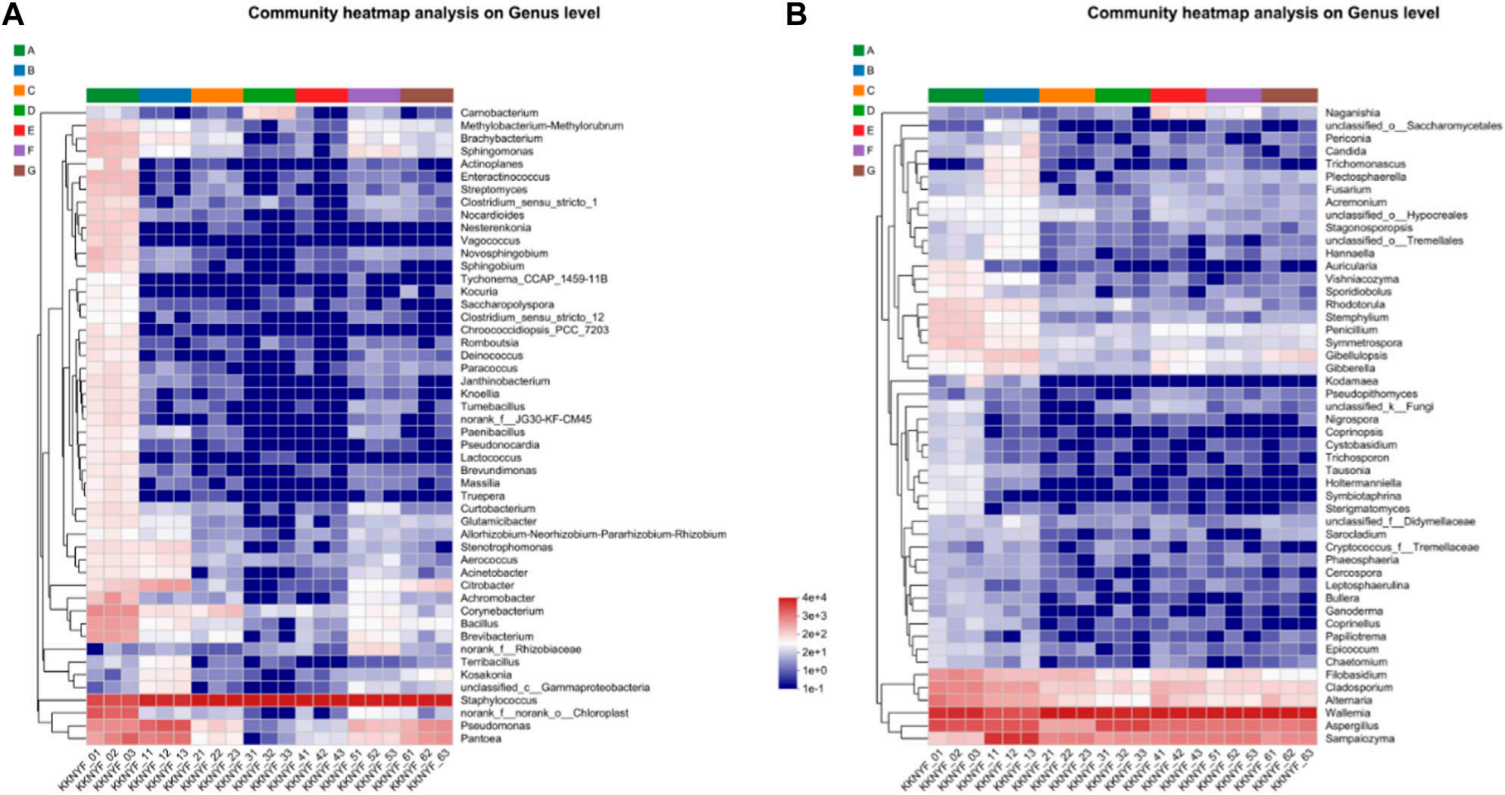
**(A)** is Heat map of microbial community distribution at the bacterial(B) genus level. **(B)** is Heat map of microbial community distribution at fungal level.

At the genus level, 15 bacterial genera and 6 fungal genera with relative microbial content greater than 0.5% were detected. The bacterial genera mainly include *Bacillus*, *Staphylococcus*, *Pseudomonas*, *Pantoea*, *Corynebacterium*, *Citrobacter*, etc. (Figures 6A, B), *Staphylococcus* accounted for the largest proportion in each sample. The fungal genera mainly include *Wallemia*, *Aspergillus*, *Sampaiozyma*, etc. *Aspergillus* accounted for the highest proportion in the first turn of the pile in the water-added group, and the rest of the samples accounted for the highest proportion of *Wallemia*. Further analysis of the community distribution heat map at the genus level showed that the community distribution in the water-added group was the most abundant and the relative abundance was high. As the fermentation progressed, the relative abundance of the dominant bacteria gradually increased, and the abundance of most bacteria decreased significantly. *Staphylococcus* and *Wallemia* are key microorganisms throughout the fermentation process (Figures 7A, B).

#### 3.6.4 Beta diversity analysis

**FIGURE 8:**
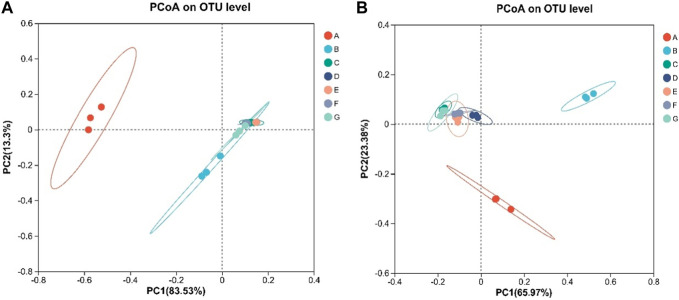
**(A)** is analysis of bacterial OUT level PCoA, **(B)** is analysis of fungal OUT level PCoA. PCoA on OTU level.

PCoA was used to compare and analyze the species diversity among the microbial communities of each sample, and to explore the similarity or difference of community composition among different groups of samples. From Figure 8, it can be seen that the control group has a large difference between the samples before fermentation and in the agricultural fermentation process. The difference between the samples after the first turning time, the second turning time and the end of the fermention of the water-added group is large. In the cocoa-added group, the samples at each stage are more concentrated, and the species diversity similarity between the samples is high. The variation trend of the samples in fungi and bacteria was basically the same, and the results were basically consistent with the variation rule of the heat map of community distribution described above. Combined with the PCoA analysis of bacteria and fungi, it can be seen that after agricultural fermentation, the microbial community in cigar leaves has changed greatly, and the traditional water-added fermentation method and the addition of cocoa medium for fermentation have played a role.

#### 3.6.5 Functional prediction of microbial communities

**FIGURE 9:**
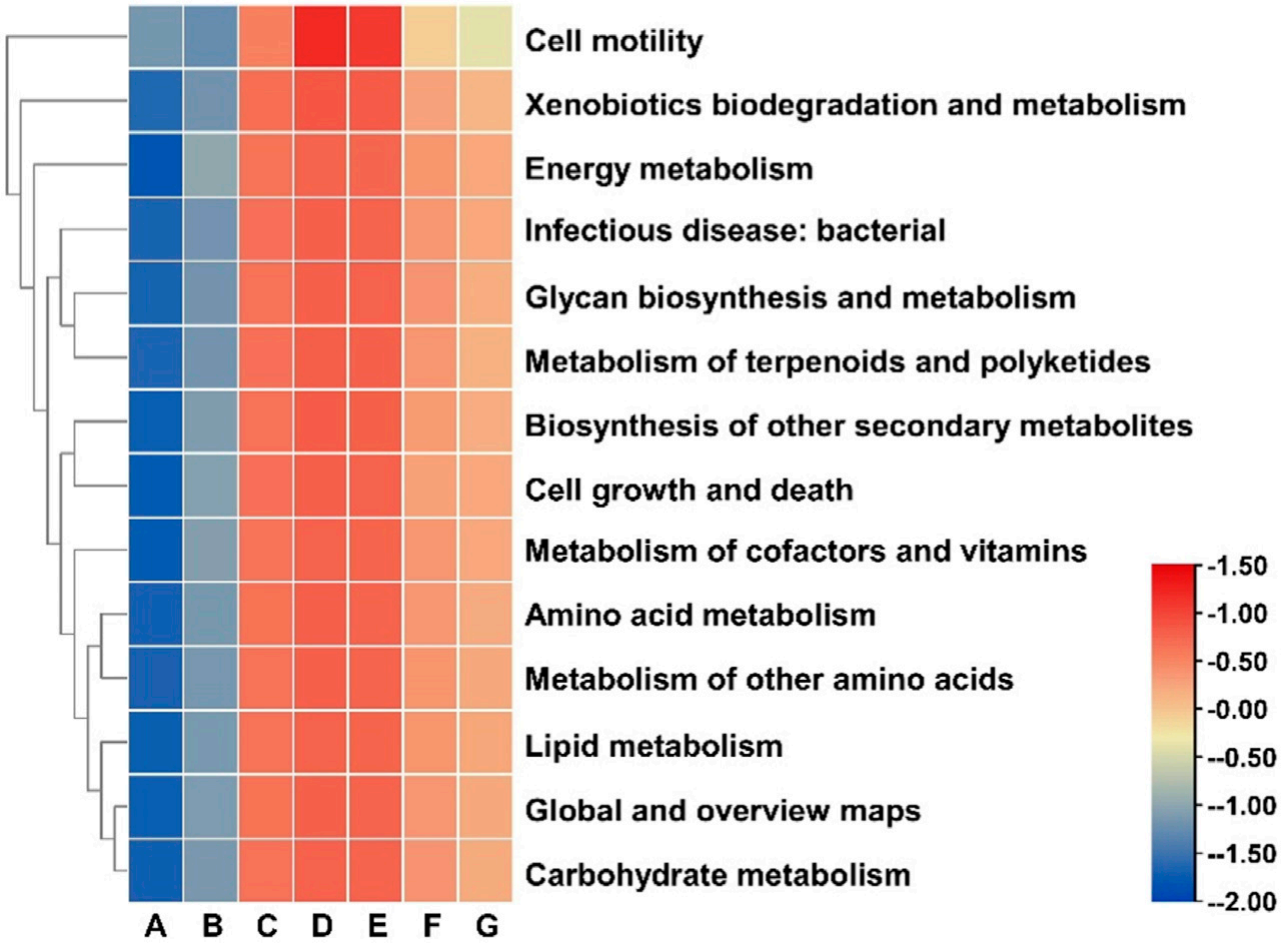
Analysis of bacterial genes in cigar leaves.

Based on the analysis of the distribution of bacterial communities in different cigar leaves, PICRUSt was used to further analyze its functional genes. A total of 22 functional categories were annotated at the levle2 for all bacterial gene sequences. The abundance of each functional category was analyzed, and 14 functional categories related to carbohydrate metabolism, amino acid metabolism, lipid metabolism, biosynthesis of other secondary metabolites, metabolism of terpenoids and polyketides, and biosynthesis and metabolism of polysaccharides were selected for analysis (Figure 9). In the water-added fermention group, the expression abundance of all functional genes was low. When water and cocoa medium were added for fermentation, at the end of the first turning time, the expression abundance of related functional genes in the water-added group and the cocoa-added groupcocoa-added group was significantly increased. The expression abundance of functional genes in the cocoa-added groupcocoa-added group was significantly higher than that in the water-added group, indicating that the addition of cocoa medium increased the expression abundance of related functional genes in cigar leaves and accelerated the fermentation process of cigar leaves. At the end of the second turning time, the expression abundance of related functional genes in the water-added group and the cocoa-added group increased, and reached the peak in this process. At the end of fermentation, the expression of related functional genes showed a downward trend, and the downward trend of the cocoa-added group was faster than that of the water-added fermention group, among which the functional genes related to cell activity decreased most significantly. It can be seen from this analysis that the addition of cocoa medium for fermentation accelerates the process of agricultural fermentation and shortens the time of agricultural fermentation. FUNGuild was used to classify the functions of fungi. From the prediction results, the function of fungi in agricultural fermentation process has low correlation with fermentation process, indicating that bacteria played a major role in the agricultural fermentation process.

## 4 Discussion

At present, the research on the fermentation of cigar leaves by adding medium is mostly concentrated in the industrial fermentatio. In this study, medium fermentation was added to cigar leaveswrapper in the agricultural fermentation stage. In the agricultural fermentation stage, the types and quantities of microorganisms retained in cigar leaveswrapper were more, so that the medium was involved in the fermentation process of cigar leaveswrapper in advance, which was conducive to improving the microbial activity in cigar leaves, making the fermentation more sufficient and improving the quality of tobacco leave (Li et al., 2016; Zhang et al., 2020). The sugars, proteases and amino acids contained in the medium are involved in the alcoholization of cigar leaveswrapper and are used by microorganisms cigar leavesto produce some beneficial metabolites.

In the process of agricultural fermentation, the conventional components of cigar leaveswrapper have changed significantly. Polysaccharides such as cellulose and starch are hydrolyzed to glucose, which is converted into aroma substances through maillard reaction and caramelization reaction to improve the aroma quantity of cigar leaves wrapper. At the same time, it can also be converted into acidic substances, which has the function of balancing the acidity and alkalinity of flue gas and reducing irritation (Xiao et al., 2010; Hu et al., 2012; Li et al., 2012; Su et al., 2017). In the early stage of fermentation, the consumption rate of sugars in the cocoa-added group was lower than that in the water-added group. In the later stage of fermentation, due to the increase of microbial activity in the cocoa-added group, the consumption rate of reducing sugars increased, which may lead to more conversion to other compounds, thereby increasing the aroma components of wrapper. Through the determination of potassium chloride content and potassium chloride ratio in cigar leaveswrapper, combined with the results of sensory evaluation, it can be seen that the addition of cocoa medium can improve the combustion of wrapper, which may be related to the fact that cocoa itself contains more potassium (Huang et al., 2022). The contents of proline and malic acid, which were positively correlated with sensory quality, were higher in the cocoa-added group than the water-added group. The contents of neutral and basic amino acids, citric acid and linoleic acid, which were negatively correlated with sensory quality, were significantly lower than those in the water-added group. It may be related to the rich oil of cocoa, which increases the fatty acid content in cigar leaves. From the analysis of the composition and content of aroma components combined with sensory evaluation, the cocoa medium may have the effect of changing the aroma structure, strengthening the aroma of coffee, cocoa and nuts, and further highlighting the aroma characteristics of cigar leaveswrapper. The bacterial diversity on the surface of wrapper during fermentation was analyzed by 16S rDNA high-throughput sequencing technology. It was found that there was little difference in microbial community structure between the water-added group and the cocoa-added group, but the dominant microorganisms in the cocoa-added group were more concentrated, and the dominant microorganisms played a more obvious role. In the early stage of fermentation, *Staphylococcus* and Corynebacterium were the main bacteria, and the relative abundance was higher than that of the water-added group. At the end of the fermentation, *Pseudomonas* and *Pantoea* were the main bacteria, and they could still maintain high activity. By analyzing the samples after the second turning of the water-added group, it was found that the microbial species and relative abundance decreased sharply, which may lead to the failure of full fermentation after the first turning, thus affecting the transformation of metabolites and affecting the improvement of tobacco quality. The study found that the relative abundance of *Staphylococcus* and Arthrobacter remained high throughout the fermentation process. It can be inferred that these two genera are the key microorganisms in the agricultural fermentation stage, which is consistent with the distribution of microorganisms in cigar leaves studied by Zheng Linlin and Zhang Lei (Zhang et al., 2021; Zheng et al., 2022). *Staphylococcus* has protease and lipase activity, which is conducive to the production of peptides, amino acids and fatty acids (Aro et al., 2010; Tabanelli et al., 2012), and plays a key role in the formation of cigar tobacco leaf flavor. Basidiomycetes is currently reported to be involved in the synthesis of alkaloids (Peng et al., 2009). Through the analysis of the expression abundance of related functional genes in the process of agricultural fermentation, it can be seen that compared with the control group, agricultural fermentation can greatly improve the microbial activity and promote the material transformation in cigar leaves, which proves that agricultural fermentation can improve the quality of cigar leaves. Adding cocoa medium fermentation can make the related functional genes express faster, play a role earlier, improve the efficiency of agricultural fermentation, and shorten the fermentation time.

Medium participation in fermentation is a common method in production, but the current research only stays in chemical composition analysis and microbial detection. After the fermentation medium is involved, it is transformed into some aroma substances or other chemicals, but after the cigar is burned, it is unknown whether some chemical components have experienced combustion and still present the taste before combustion. Therefore, the research tested the changes of chemical composition and microbial community of cigar leaves with and without adding medium, combined with artificial evaluation, in order to prove that it is useful for fermentation. Through the current research, it can only be proved that some oil, protein and other substances in the cocoa medium have an impact on the richness of the fragrance, but the analysis of the fermentation to combustion process has not been able to connect in series.At present, there are still some quality defects in the cigar leaves treated by conventional agricultural fermentation. If the medium fermentation can be added in advance in the agricultural fermentation stage to strengthen the treatment effect in the agricultural fermentation stage, it is beneficial to improve the compatibility of raw materials in the product formula, improve the competitiveness of domestic cigar leaf raw materials, improve the proportion of domestic cigar leaves in Chinese cigars, and save the production and time cost of industrial enterprises. There are few studies on the application of media in the stage of agricultural fermentation. This study preliminarily explored the effects of adding media fermentation in the stage of agricultural fermentation on the chemical composition, microbial community and functional gene expression level of cigar leaves, which laid a foundation for improving the quality of cigar cigar leaves by using media fermentation in the future.

## 5 Conclusion

In this paper, the changes of chemical components and microorganisms on the surface of cigar leaves were studied by adding cocoa medium in agricultural fermentation stage. The experimental results showed that the addition of medium promoted the conversion of sugars to aroma substances, and the addition of medium fermentation was beneficial to increase the potassium content and potassium-chlorine ratio of cigar leaves and improve the flammability of cigar leaves. At the same time, the content of proline, malic acid and other substances positively correlated with sensory quality increased, and the total amount of aroma components in cigar leaves increased significantly, especially olefins and acids, which changed the structural composition of some aroma substances in cigar leaves. The results of high-throughput sequencing of microorganisms showed that bacteria played a major role in the fermentation process. The addition of media directly affected the dominant flora in cigar leaveswrapper, improved microbial activity, and accelerated the process of agricultural fermentation. This study preliminarily revealed the mechanism of cocoa medium to improve the quality of wrapper, and also provided more theoretical support for the application of new medium in agricultural fermentation stage.

## Data Availability

The original contributions presented in the study are publicly available. This data can be found here: https://www.ncbi.nlm.nih.gov/bioproject/PRJNA1002093/.
